# Olfaction-Related Factors Affecting Chemosensory Dream Content in a Sleep Laboratory

**DOI:** 10.3390/brainsci11091225

**Published:** 2021-09-16

**Authors:** Lenka Martinec Nováková, Monika Kliková, Eva Miletínová, Jitka Bušková

**Affiliations:** 1 National Institute of Mental Health, Topolová 748, 250 67 Klecany, Czech Republic; monika.klikova@nudz.cz (M.K.); eva.miletinova@nudz.cz (E.M.); jitka.buskova@nudz.cz (J.B.); 2 Department of Psychology and Life Sciences, Faculty of Humanities, Charles University, Pátkova 2137/5, 182 00 Prague 8-Libeň, Czech Republic; 3 Third Faculty of Medicine, Charles University, Ruská 87, 100 00 Prague 10-Vinohrady, Czech Republic

**Keywords:** chemoreception, dream, expectancy, mental sleep experience, odour awareness, odour identification, rapid eye movement, recall, sleep, smell

## Abstract

Mental activity in sleep often involves visual and auditory content. Chemosensory (olfactory and gustatory) experiences are less common and underexplored. The aim of the study was to identify olfaction-related factors that may affect the occurrence of chemosensory dream content. Specifically, we investigated the effects of all-night exposure to an ambient odour, participants’ appraisal of their current olfactory environment, their general propensity to notice odours and act on them (i.e., odour awareness), and their olfactory acuity. Sixty pre-screened healthy young adults underwent olfactory assessment, completed a measure of odour awareness, and spent three nights in weekly intervals in a sleep laboratory. The purpose of the first visit was to adapt to the experimental setting. On the second visit, half of them were exposed to the smell of vanillin or thioglycolic acid and the other half to an odourless control condition. On the third visit, they received control or stimulation in a balanced order. On each visit, data were collected twice: once from the first rapid eye movement (REM) stage that occurred after 3 a.m., and then shortly before getting up, usually from a non-REM stage. Participants were asked to report the presence of sensory dream content and to assess their current olfactory environment. Neither exposure, nor participants’ assessments of the ambient odour, or olfactory acuity affected reports of chemosensory dream content but they were more frequent in individuals with greater odour awareness. This finding may have implications for treatment when such experiences become unwanted or bothersome.

## 1. Introduction

Sleep, which manifests behaviourally as being perceptually disengaged and unresponsive to the environment, may seem to observers to be a more or less uniform state of inactivity. Yet while perception is reduced as sensory thresholds increase (and sensory sensitivity declines) during the transition to sleep [1], it is not a homogeneous state of cerebral and mental quiescence. In sleep, we experience mental elaboration that is not directly observable and is only reported upon awakening in a different physiological state. In scientific language, this experience is referred to as ‘sleep mentation’, ‘mental sleep activity’, or ‘mental sleep experience’ (MSE), while in common speech it is known as ‘dreaming’ [2]. Nevertheless, the three more general scientific terms are preferred because the term ‘dream’ may imply a particular sort of sleep experience.

Sleep mentation has traditionally been categorised as either dreamlike or thought-like. MSE content referred to as ‘dreamlike’ is particularly rich in (predominantly visual) imagery, tends to be bizarre, hallucinatory, emotionally charged, and have a complex story-like narrative organisation. It became associated with the rapid eye movement (REM) sleep stage [3,4,5] and is viewed by some researchers as synonymous with ‘dreaming’ [5]. The thought-like type of MSE, on the other hand, is more verbal, poorer in imagery, briefer, and more fragmented, and is viewed as being linked to non-REM (NREM) sleep [6]. Still, the notion of systematic or strong links between the particular sleep stages and specific MSEs has been challenged [7] and the magnitude of differences between the dreamlike and thought-like experiences disputed [5,8]. It is thus probably best to conceptualise sleep mentation as occurring along a continuum that ranges from a dreamlike content typical of the REM all the way to the thought-like experiences characteristic of early NREM sleep. In the present study, ‘dreaming’ is understood as denoting any MSE, regardless of its nature and irrespective of the sleep stage in which it is assumed to originate.

As noted above, reports of mental activity during both REM [9] and NREM sleep [10] may contain references to various sensory experiences, most commonly (audio)visual [11,12,13]. In contrast, MSEs with a chemosensory (olfactory or gustatory) component seem considerably less frequent, as Supplementary Table S1 suggests. Nevertheless, some methods of data collection, including retrospective questionnaires, may underestimate the actual rate of occurrence of smell and taste in dreams [14]. On the other hand, home diaries or logs kept by participants over various periods of time can increase dream recall rates and hence also the frequency of reports of chemosensory experiences as people learn to keep records of their MSEs [14].

Yet another option is to collect MSE reports in a sleep laboratory upon spontaneous or forced (instrumental) awakening from a predefined sleep stage, usually the REM [15,16,17,18,19]. The main reason REM reports are of special interest is because it has been found that this sleep produces higher recall rates than the NREM and longer, more perceptually vivid, motorically animated, and emotionally charged reports, which are moreover less related to waking life and current personal concerns. Still, chemosensory content is rare even in REM reports. For instance, McCormick, Nielsen, Ptito, Hassainia, Ptito, Villemure, Vera and Montplaisir [15], who examined 80 reports obtained from eight participants only found three mentions (4%) of chemosensory content. Similarly, out of 39 reports there was only a single explicit mention (3%) of olfactory content in a study carried out by Schredl, Atanasova, Hoermann, Maurer, Hummel, and Stuck [18]. The authors asked two independent raters, who had not been involved in data collection, to identify dream elements potentially associated with smell. No such elements were found. These findings were further corroborated by Schredl, Hoffmann, Sommer, and Stuck [19].

Despite being rather uncommon, chemosensory content in sleep mentation deserves closer attention because it occurs more frequently in connection with various neurological and psychiatric disorders [20,21], such as migraine [22,23], epilepsy [24,25], or post-traumatic stress disorder [26]. It is therefore important to ask under what circumstances it occurs in healthy individuals. The present study focuses on various olfaction-related factors. One such potential factor may be the ambient atmosphere. Early dream researchers used straightforward techniques of odour presentation, such as spraying odorants on the sleeping person’s pillow, to explore whether being exposed to odour can modify sleep mentation and its content [27]. Current scientific studies, in which simple odour administration techniques have been superseded by sophisticated olfactometry, have corroborated the notion that smells can indeed affect some aspects of sleep mentation [16,18,28]. Even so, it is rare for olfactory stimuli to be incorporated into MSEs [18,19,29], be it directly, so that individuals report dreams about the stimulus used for exposure, or indirectly, that is, by triggering the appearance of stimulus-related themes. While delivering olfactory stimuli to the nose via an olfactometer addresses the issue of olfactory adaptation and habituation, which develops when smelling ambient odourised air [30], it is not particularly helpful for understanding the chemosensory dream content that appears when sleeping in one’s own bedroom. For instance, Solms [20] presents cases of patients who complained about unpleasant olfactory sensations incorporated into their MSEs. To help explain the occurrence of such rare but potentially bothersome dream content, it seems more appropriate to investigate the contribution of odour exposure to chemosensory dreaming under environmentally more realistic circumstances.

It is nonetheless possible that current olfactory environment bears relatively little relevance to chemosensory content in dreams. As Johnson [31] notes, citing Jellinek [32], the changes in physiology, psychology, and behaviour we experience in the presumed or actual presence of odours cannot be simply attributed to some characteristic of the ambient atmosphere (e.g., to the physicochemical properties or hedonic valence). They seem to be the result of complex mechanisms which are yet to be fully elucidated. For example, according to one account, which is compatible with other proposed explanations, rather than merely responding to our olfactory surroundings we act upon the beliefs and expectations related to particular odours. As demonstrated by e.g., Knasko et al. [33], even in absence of any experimental odour, erroneous beliefs about its presence may produce various psychological effects. Such effects should be all the more evident when an individual is consciously aware of an odour that is actually present, although one sleep/dream study reported evidence to the contrary [29]. Central to this debate is appreciation of the difference between an odour, that is, the percept created in an individual’s nervous system, and the odorant as a physicochemical entity [34]. In line with this, it has been shown that neural activation during olfactory stimulation reflects what subjects thought they were smelling rather than the actual stimulus [35]. This is why subjects’ subjective appraisal of the ambient atmosphere should always be considered along with the objective properties or generally agreed-upon qualities of the stimuli. In our study, we have therefore also tested the effect of whether any odour was perceived or not on a given occasion.

Besides the between-subject variation in evaluation of current olfactory environment, we were also interested in the effects of some more general olfaction-related characteristics. A number of previous studies showed how individual tendencies in the affective and cognitive processing of chemosensory stimulation modulate the effects which ambient atmosphere has on our functioning and wellbeing [36,37,38,39]. One such factor is olfactory acuity [36]. In this study, it was operationalised as the ability to identify odours, which has been previously shown to correlate with self-assessments of olfactory dreaming [40,41].

People do, however, also exhibit a wide range of olfactory behaviours that cannot be captured by olfactory tests and have been subsumed under the umbrella term ‘odour awareness’ [42]. Odour awareness manifests itself in the way individuals interact with their olfactory environment and use their sense of smell in everyday contexts, whereby higher levels of odour awareness have been linked to a greater tendency to experience chemosensory content in sleep mentation [41,43].

In order to assess the impact of all-night odour presence and investigate the individual appraisal of the ambient atmosphere, odour identification, and awareness of chemosensory dream content experienced under experimental conditions, we invited 60 pre-screened healthy young adults to undergo olfactory assessment, complete a measure of odour awareness, and spend three nights in weekly intervals in a sleep laboratory. After the first adaptation night, on the second and third visit we exposed one half of the volunteers to the smell of vanillin or thioglycolic acid and the other half to an odourless control condition in a balanced order. Odour exposure was continuous because this study sought to examine these effects under experimental conditions that would be as close to a domestic environment as possible. On each visit, data were collected twice: once from the first rapid eye movement (REM) stage that occurred after 3 a.m., and then shortly before getting up, usually from a non-REM stage. In most participants, this produced six repeated measures of dream presence or absence, eventual dreams’ sensory content, and evaluations of current olfactory environment, which were collected immediately upon each awakening.

## 2. Materials and Methods

### 2.1. Participants

The participants were 60 healthy young adults (37 males, mean age 23.43 ± 3.80 SD years, range 19–35 years) who had been pre-screened with a set of questionnaires and inventories for the absence of (i) psychiatric, neurological, and other conditions influencing the sense of smell [44] or sleep [45,46], (ii) problems in daily life due to olfactory impairment [47,48], (iii) mild or more severe depressive tendencies [49], (iv) tobacco dependence beyond light smoking [50], and (v) alcohol dependence beyond moderate drinking [51]. The exclusion criteria were thus as follows: (i) any current psychiatric, neurological, or other condition that might affect olfaction or sleep, or a history thereof, (ii) a score below a cut-off point of 38.5 on the Questionnaire of Olfactory Disorders–Negative Statements (QOD-NS) that would be potentially indicative of absence of normosmia [52], (iii) a score on the Beck Depression Inventory II (BDI-II; [49,53]) above the minimal range of 0–13 points [54], (iv) smoking over 90 cigarettes a month, (v) drinking more than one or two drinks a day in women and men, respectively, where a ‘drink’ is 0.33 L of beer, 0.2 L of wine, or 0.02 L of liquor, (vi) history of substance abuse, particularly illicit drug use, (vii) pregnancy or breastfeeding in women. Participants further had to meet the following inclusion criteria: age 19–35 years (to reduce potential confounding influences of age-related physiological variation in the sense of smell and sleep), use of hormonal birth control in women in order to minimise olfactory fluctuations across the menstrual cycle, being able to recall at least one dream a week, and generally experiencing dreams that are rather emotionally intense.

Recruitment of participants was conducted via posts advertising the study on the website and Facebook page of the National Institute of Mental Health (NIMH), as well as social media of the Faculty of Humanities, Faculty of Science, the Third Faculty of Medicine, Faculty of Education, Charles University, and the Faculty of Agrobiology of the Czech University of Life Sciences in Prague. Digital and paper posters were put up in the lobbies of the NIMH and the faculties. Individuals interested in the study received an e-mail link to a set of screening questionnaires and inventories administered through the Qualtrics platform (Qualtrics, Provo, UT, USA). Those who complied with the exclusion and inclusion criteria were examined by a physician (EM) before the first night. Descriptive statistics of the final sample are given in Table 1.

Participants received a financial compensation of 1500 CZK (app. 58 EUR).

### 2.2. Questionnaires

#### 2.2.1. Health- and Sleep-Related Screening Inventory

The set of screening questionnaires and inventories e-mailed to those interested in the study started with a brief instrument to evaluate their medical history, sleep habits, and use of substances affecting sleep (e.g., caffeine, nicotine, alcohol, illicit drug use). This data was used to evaluate compliance with exclusion criteria i, iv, v, and vi.

#### 2.2.2. Questionnaire of Olfactory Disorders—Negative Statements

Absence of problems in daily life due to olfactory impairment (exclusion criterion ii) was assessed with a previously validated, short modified version of the Questionnaire of Olfactory Disorders—Negative Statements (QOD-NS) [47,48,55]. It has been suggested that this questionnaire is a useful tool for olfactory screening and moreover, it correlates with Sniffin’ Sticks scores, especially regarding those related to odour discrimination [52]. It consists of 17 negative statements describing various difficulties in everyday life related to impaired olfaction, such as ‘From waking up until bedtime, I am aware of my difficulties with smelling’, ‘Difficulties with smelling impair my appreciation of drinks and foods’, ‘Because of difficulties with smelling, I am scared of getting exposed to certain dangers (e.g., gas, rotten food)’. By answering each item on a scale from 0 to 3, respondents indicate the extent to which they are facing that particular issue, with higher scores suggesting less difficulty. A total score of 51 indicates therefore no problems in daily functioning related to olfaction. Scores below the cut-off point of 38.5 were regarded as possibly indicative of non-normal olfaction [52].

#### 2.2.3. Beck Depression Inventory II

Depressive tendencies (exclusion criterion iii) were screened using the 21-item BDI-II [53], published in Czech by Preiss and Vacíř [49]. In this questionnaire, respondents answer items related to different symptoms or attitudes by selecting one of four graded statements. These are weighted from 0 (not present) to 3 (severe), with higher scores indicating greater severity. Items cover sadness, pessimism, past failure, loss of pleasure, guilty feelings, feelings of punishment, self-dislike, self-criticalness, suicidal thoughts or wishes, crying, agitation, loss of interest, indecisiveness, feelings of worthlessness, loss of energy, changes in sleeping patterns, irritability, changes in appetite, difficulties concentrating, tiredness or fatigue, and loss of interest in sex. Weights corresponding to the selected statements are then added to obtain a total score, which can range between 0 and 63. Higher total scores indicate greater depressive tendencies. For the purpose of our study, only low-scoring respondents (with 0–13 points) were regarded as eligible for inclusion.

#### 2.2.4. Odor Awareness Scale

Participants’ propensity to notice smells and act on them in everyday contexts was evaluated using the 32-item Odor Awareness Scale (OAS) [42]. It was translated into Czech by LMN and Jaroslava Varella Valentova and used in several prior studies [56,57]. Items pertain to the tendency to pay attention to odours (‘When you visit someone else’s house, do you notice how it smells?’), to seek olfactory stimulation (‘Do you sniff a new book?’), and to the effects of chemoreception on mood (‘Do you feel cheerful or happy when you pick up a pleasant odour in the air?’), self-grooming (‘The smell of smoke or food is still lingering in your clothes from the night before. Do you put on new clothes because of the smell?’), attractivity judgments (‘When someone has an unpleasant body odour, does that make you find him or her unattractive?’), or food preferences (‘Does the smell of food sometimes put you off it?’). Participants respond to 30 of the 32 items by choosing one of five categories (i.e., for frequency, the options are ‘always’, ‘often’, ‘sometimes’, ‘seldom’, and ‘never’). Greater degree, probability, or frequency are assigned higher scores. Individual scores are added and the total score can range between 32 and 158. Respondents with higher scores are considered more olfaction oriented.

#### 2.2.5. Dream Inventory

To collect information about participants’ sleep mentation and its sensory content, participants completed a custom-designed inventory immediately following each awakening. They were first asked whether they had been dreaming (‘Have you been dreaming?’, yes/no). Affirmative response to the item prompts a question about the sensory content (olfactory, gustatory, visual, auditory, somatosensory, kinaesthetic, and nociceptive; select all that apply). The instrument included several other items not used in the present study (see Martinec Nováková, et al. [58] for details).

#### 2.2.6. Olfactory Assessment

To ascertain normal olfactory function, which we assumed on the basis of their QOD-NS scores, participants were administered a Czech translation of the German version of the University of Pennsylvania Smell Identification test (UPSIT) [59]. This standardised microencapsulated (‘scratch and sniff’) test consists of 40 stimuli released by rubbing an odourised field with a pencil tip. Each stimulus comes with a set of four possible labels (one target and three distractors). Correct choices score one point each and are added, so that the total score can range between 0 and 40 points. It has been reported that the test–retest reliability of UPSIT exceeds 90% [60]. The test can be completed in about 20 min. The labels were translated into Czech by LMN and Anna Kernerová.

Olfactory threshold was not assessed due to various methodological challenges that could not be addressed in the present study, such as the need for repeated testing to obtain reliable results [61,62]. Nevertheless, based on the findings of Mattos, Schlosser, Storck and Soler [52] who observed that impaired odour threshold is associated with decreased QOD-NS scores even with normal odour identification, we were able to infer intact olfactory sensitivity (i.e., threshold scores not significantly different from age-adjusted normative values). Considering the participants’ age, we assumed a preponderance of the juvenile olfactory phenotype (see for a definition, Mazzatenta, et al. [63]).

### 2.3. Olfactory Stimulation

#### 2.3.1. Olfactory Stimuli

Each participant was stimulated with either the odour of vanillin or that of thioglycolic acid. Vanillin was purchased from Chemnovatic as 10% vanillin (CAS No. 121−33−5) and diluted in pharmaceutical propylene glycol (CAS No. 57−55−6) to 5%. Thioglycolic acid was obtained from Sigma Aldrich and diluted in distilled water to 2.5%. Vanillin is described as having a pleasant odour [64,65] and has been found to only weakly stimulate the trigeminal nerve (CN V) [66]. Thioglycolic acid has a repulsive, somewhat sour odour [64,65]. The degree to which thioglycolic acid is capable of producing trigeminally-mediated sensations of nasal pungency is unclear, but no nasal irritation has been reported even at concentrations higher than those employed in the present study [67]. To determine odour intensities optimal for olfactory stimulation, we prepared several solutions of different concentrations for both of the stimulus odours. A gauze pad was then sprayed with app. 2 mL of a particular solution and placed in a common aroma diffuser to disperse the odour in one of the bedrooms in the sleep laboratory (10 m^2^). After eight hours of continuous room odourisation, ten naïve normosmic individuals were asked to enter the bedroom to rate the ambient atmosphere for odour pleasantness and intensity. All raters could smell both odours. Similar odour intensities (median: 6 for vanillin, 5.5 for thioglycolic acid on a 10-point Likert scale) were achieved with 5% vanillin and 2.5% thioglycolic acid. As intended, the smell of vanillin (median hedonic rating 6.5 out of 10) was rated as more pleasant than that of thioglycolic acid (3.5).

#### 2.3.2. Odour Presentation

Stimuli were always freshly prepared immediately prior to the beginning of each session as per the procedure described above. Odour was dispersed with a commercially available diffuser, Otello (Mr & Mrs Fragrance, Joy Fragrances, Busto Arsizio, Italy). On the adaptation nights and for control, i.e., non-stimulation, sessions, the diffuser contained only a clean, non-odourised pad. The device was located under the bed, near the bedhead. In order to mimic conditions typically of home environment, odour exposure was continuous, starting at 10 p.m. and ending around 6:30 a.m., depending on the morning waking time. Volunteers were encouraged to assess the ambient atmosphere upon each awakening. First, they were asked whether they could smell an odour. An affirmative response prompted questions about its pleasantness, intensity, and familiarity.

### 2.4. Video-Polysomnography

On each of the three visits to the sleep laboratory, nocturnal (10 p.m. to app. 6:30 a.m.) video-polysomnographic (v-PSG) recordings were obtained with BrainScope equipment (M&I, Prague, Czech Republic). The recordings included electroencephalography (EEG; F3/A2, F4/A1, C3/A2, C4/A1, O1/A2, and O2/A1 leads), electrooculography (EOG), mental and tibialis anterior electromyography (EMG), electrocardiography (ECG), monitoring of nasal–oral air flow, oropharyngeal sounds, thoracic and abdominal efforts (via belts), pulse oximetry, and synchronised video and audio monitoring. Scoring of sleep stages, arousals, periodic leg movements, respiratory events, and EMG activities was carried out by a trained evaluator (JB) in compliance with international criteria [68]. The evaluator was blind to the distribution of volunteers in randomisation groups. Because the first visit was meant to adapt participants to the experimental setting, only data from the subsequent two sessions were entered into subsequent analyses.

### 2.5. Procedure

Volunteers deemed eligible based on the pre-screening were asked to make an appointment with the in-house physician to undergo a physical examination at the sleep laboratory. Most appointments took place in the afternoon on the day of their first, i.e., adaptation, session. Upon their check-in at the sleep laboratory at about 7 p.m., the volunteers received full instructions and were familiarised with the v-PSG procedures and with the measures they would be asked to complete upon awakening. Then they took the smell identification test (UPSIT), assessed their odour awareness (using OAS), and were fitted with electrodes and sensors. Subsequently, they were given time to relax and entertain themselves as they pleased as long as they remained at the premises of the NIMH. Shortly before 10 p.m., volunteers were asked to turn off their electronic devices and instructed to keep the lights switched off. The v-PSG was set up by one of the nurses employed at the sleep laboratory. Recording started when researchers finished preparing a participant for the night. Five hours into the recording, researchers began to scan the records in real time for characteristics of the REM stage. They were looking for simultaneous occurrence of EEG desynchronisation, absence of spindles or K-complexes, atonia, rapid eye movements, and behavioural appearance of sleep. When these characteristics persisted for five minutes, the volunteer was awakened, temporarily disconnected from the v-PSG, asked to complete the dream inventory, and instructed to evaluate the ambient atmosphere. This usually took no longer than five minutes. V-PSG recording was then resumed and lasted until the morning waking time. Before getting up, the volunteer completed the same measures once again, this time for the sleep episode(s) which followed the first instrumental awakening.

In total, there were 240 awakenings, 228 of which could be submitted for analysis. In 12 awakenings from nine participants, some data were missing due to late (i.e., post−6:30 a.m.) occurrence of the five-minute REM period, failure to complete the session on the volunteer’s part, or technical issues.

On the first morning, volunteers received sleep diary worksheets to record their sleep routine over the next two weeks and actigraphs to verify the records using activity-based sleep-wake monitoring. Ongoing compliance with participation criteria of good health and absence of problems with the sense of smell was ascertained by contacting participants by phone shortly before their second and third visits. Procedures followed on the next two visits were identical to those described above, except for assessments of odour identification and awareness, which were not re-evaluated on the second and third night. The data were collected between February 2017 and April 2018.

### 2.6. Statistical Analysis

**Figure 1:**
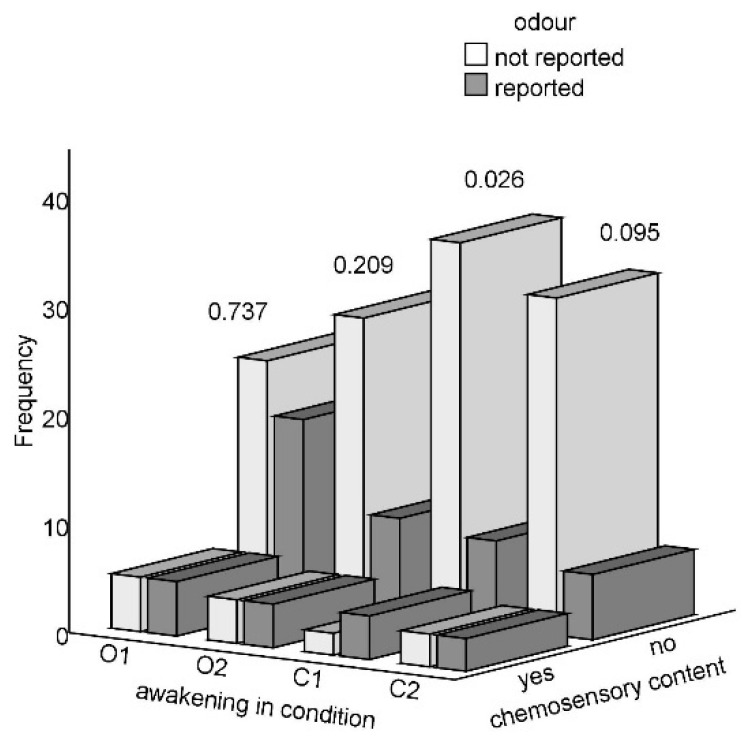
Absolute frequencies of sleep mentation reports involving or lacking chemosensory content across the four awakenings, categorised according to odour detection (hit/miss; in O1 and O2) or belief about odour perception (false alarm/correct rejection; in C1 and C2); *p*-value for a chi-square test is shown for each awakening.

Kruskal-Wallis ANOVAs, Mann-Whitney U, Wilcoxon signed rank, Pearson’s chi-square tests, and Pearson’s product-moment correlations were run using IBM SPSS 24.0 software [69], which was also used to produce the plot shown in Figure 1. SAS University Edition [70] was employed to fit the Generalised Estimating Equations (GEEs) and logistic regression models. Mid-p tests [71] were performed online using OpenEpi [72]. We examined the data for normality and outliers with histograms, skewness and kurtosis z-scores, and Shapiro-Wilk’s W tests. Since these analyses revealed non-normality, we preferred non-parametric tests wherever possible.

Variation among randomisation groups in continuous variables was explored with Kruskal-Wallis ANOVAs and Mann-Whitney U tests, while Pearson’s chi-square tests were run on categorical data. We used Fisher’s exact probability tests for 2 × 2 contingency tables with low cell counts and Freeman-Halton extension for 2 × 3 and 2 × 4 contingency tables [73]. Effect sizes (r) for Mann-Whitney U tests were calculated by dividing the z-score of test statistic by the square root of *n* [74]. Effect sizes for the chi-square tests are represented by the odds ratio.

In order to model effects of the condition (odour/control), participants’ appraisal of actual olfactory environment (odour reported/not), odour awareness (OAS score), and olfactory identification (UPSIT score) on reports of chemosensory content in sleep mentation across the four awakenings, we fitted a GEE model. It is an extension of the semi-parametric method for longitudinal data that makes minimal distributional assumptions. It estimates parameters using quasi-likelihood [75,76]. To account for correlations between observations (which were due to the fact that we had four awakenings per participant) and the binary nature of the outcome (chemosensory content being assessed as either present or absent), we fitted a GEE model for binary correlated data with a logit link function. This was done using the PROC GENMOD procedure in SAS (see e.g., [77] for a tutorial). Odds ratios (ORs) are a good estimate of relative risk when the prevalence of an event is low–as it was in our study [78]. Odds ratios express the effect of each individual predictor in the model adjusted for the other predictors.

To assess goodness-of-fit, we used the quasi-likelihood information criterion (QIC), which is a modification of the better-known Akaike information criterion (AIC) for GEEs [79]. Model fit was further assessed visually by producing calibration plots for each condition (odour vs. control, first vs. second awakening). Marginal R^2^ [80,81] was calculated using the SAS macro %SelectGEE [82]. Marginal R^2^ can be interpreted as the portion of variance in the outcome explained by the model. Because GEEs do not produce predicted probabilities, these were computed using a penalised likelihood estimation, also known as the Firth method [83]. To enable a comparison between our findings and results reported by previous studies, conversions among effect size measures were carried out according to Borenstein, et al. [84].

### 2.7. Data Availability

See Supplementary Table S2 for the data analysed in GEEs.

## 3. Results

### 3.1. Descriptive and Exploratory Statistics

#### 3.1.1. Ratings of Olfactory Stimuli

As shown in Supplementary Table S3, in the odour condition on the first awakening (O1) significantly more people correctly detected vanillin (*n* = 19, 63.3%) than thioglycolic acid (*n* = 9, 32.1%). On the second awakening (O2), the smell of vanillin was also more readily perceptible than that of the thioglycolic acid: 13 (44.8%) vs. 5 (17.9%) reports, respectively. Importantly, thioglycolic acid was rated as significantly less pleasant than vanillin on both occasions (moderate effect size), while their intensity and familiarity ratings did not differ.

#### 3.1.2. Variations between Randomisation Groups

To test the differences between sleep stages from which the volunteers were awakened while being stimulated with vanillin vs. thioglycolic acid on the second vs. third visit, due to low cell counts we had to pool NREM data to form a single group. For the same reason, we used the Freeman-Halton extension [73] of the Fisher exact probability test for a 2 × 4 contingency table. Even so, analyses still could not be meaningfully performed for the first awakenings, which were from REM stage in app. 90% of participants (see also Table 2). For the second awakenings, sleep stages did not significantly differ between the randomisation groups regardless of whether they were analysed according to the visit (visit 2: χ^2^(3) = 3.11, *p* = 0.375; visit 3: χ^2^(3) = 1.45, *p* = 0.694) or the condition (odour: χ^2^(3) = 2.60, *p* = 0.457; control: χ^2^(3) = 2.80, *p* = 0.424). Comparisons of dream recall between the randomisation groups were not meaningful due to low cell counts, but visual inspection suggested similar frequencies of positive reports regardless of the randomisation group. In subsequent analyses, participants were therefore treated as a homogeneous group and their randomisation status was disregarded.

### 3.2. Occurrence of Chemosensory Content in Sleep Mentation and Contributing Factors

Table 2 and Figure 1 show that the frequency of chemosensory dreaming across the four awakenings was low irrespective of the condition. The effect sizes for comparisons between the odour and control condition (expressed as conditional maximum likelihood estimates of odds ratio, OR) were mostly small and did not reach the minimum effect size deemed to represent a ‘practically’ significant effect [85]; see Table 2 for details. In particular, chemosensory content was reported by three to seven participants on each occasion. Of the 27 participants who could recall a ‘dream’ on all four occasions, 21 (77.8%) reported no MSE featuring smell, five people (18.5%) reported olfactory content only once, and just one person’s (3.7%) sleep mentation always involved smelling something. Similarly, absence of gustatory content was reported by 20 people (74.1%), three people (11.1%) had one such experience, and two and three reports involving taste were provided by two people each (7.4%). From Table 2 it is also apparent that volunteers were significantly more likely to smell an odour during exposure than in the control condition.

Nevertheless, a joint exploration of reported smell and taste content and the volunteers’ appraisal of the ambient atmosphere revealed two outliers. One person reported olfactory and gustatory content four and three times, respectively, and another mentioned smell and taste one and three times, respectively. Both believed they could smell an odour upon each of the four awakenings. These two cases were therefore excluded from the subsequent GEEs. Because chemosensory dreaming involved a combination of olfactory and gustatory content in 50% of cases, reports of smell and taste dreaming were merged into a single variable of chemosensory content (present/absent).

Results of the GEE model for correlated binary outcomes, detailed in Table 3, revealed that neither the condition (odour/control) nor the participants’ appraisal of current olfactory environment upon awakening (odour reported/not reported) had a significant effect on reports of chemosensory content: OR = 1.86, 95% confidence limits (95% CL) [0.69; 5.00], χ^2^ = 1.50, *p* = 0.22, and OR = 1.67 [0.61; 4.56], χ^2^ = 1.01, *p* = 0.31, respectively. On the other hand, such reports were significantly more likely in participants who indicated a greater propensity to notice odours and act upon them. After adjusting for correlated outcome data and controlling for the other effects, the estimated change in the odds of reporting chemosensory content for a one-point increase in the Odor Awareness Scale (OAS) score was OR = 1.05 [1.02; 1.08], χ^2^ = 13.38, *p* <0.001. In other words, there was a 5% increase in the odds of reporting chemosensory content for a one-point increase in OAS. For a 10-point increase in OAS with a range of 63–127, this gives a 62% increase in the odds of experiencing smell or taste in sleep mentation upon any given awakening. Odour identification had a negative but statistically non-significant relationship with chemosensory content (OR = 0.91 [0.78; 1.06], χ^2^ = 1.55, *p* = 0.21). Predicted probabilities of reporting chemosensory content upon individual awakenings, given in full in Supplementary Tables S4–S7, revealed that there was a 7–26% chance that participants with median OAS and UPSIT scores would report smelling or tasting in their MSEs irrespective of whether odour presence was reported. Nevertheless, the whole model only explained 6% of variance in the outcome (marginal R^2^ = 0.06). Interaction between the condition and participants’ assessment of current olfactory environment was not included in the model because it increased the QIC. Nonetheless, an alternative model which included this interaction yielded similar results, with the interaction being statistically non-significant (*p* > 0.40).

## 4. Discussion

The aim of the study was to identify olfaction-related factors that may influence reports of chemosensory (olfactory and gustatory) content in sleep mentation in a sleep laboratory. In line with previous research, we found that the occurrence of chemosensory dreaming was uniformly low irrespective of stimulation. The percentage of MSEs involving smelling and tasting was nonetheless slightly higher than previously observed in other laboratory studies [18,19]. In those studies, the presence of chemosensory content was assessed by researchers, not by the participants themselves. Having participants indicate whether their sleep mentation involved smell or taste may be a potential source of bias because some participants might believe that chemosensory dreaming, being both intriguing and rare, is a desirable attribute. Such belief may have affected responses of the two volunteers whose reports we excluded from the analyses as outliers. These two participants might have also felt that odour detection was always expected, even when in reality no odour was presented. There were another three participants who were also convinced that they could smell an odour upon each awakening, but they did not report any chemosensory dreaming.

One potential explanation of why odours influence our physiology, mood, and cognition is that participants hold various prior beliefs or have expectations regarding the effects of odours, as reviewed by Johnson [31], citing Jellinek [32]. In the present study, except for the two outliers, participants’ beliefs about odourisation, whether correct or erroneous, did not appear to produce reports of chemosensory dreaming. For example, a false belief about the presence of an odour upon either awakening in the control condition was shared by 13 and 11 participants, respectively, but olfactory content was reported only in three and two instances, respectively (see Table 2 for details). The rates of chemosensory content recall were similarly low in the odour condition, where more people (correctly) reported odour presence. According to the expectation-based explanation, odours should affect sleep mentation predominantly or only when participants are consciously aware of their presence (on the other hand, cf. e.g., Lorig, et al. [86], Kuroda, et al. [87] for evidence that conscious olfactory experience is not instrumental in bringing about odour-induced effects). This was not the case in the present study because the generally low frequencies of chemosensory dreaming seemed unrelated to participants’ appraisal of the ambient atmosphere (see also Figure 1). Future studies should nevertheless conduct post-participation explorations of volunteers’ expectations about the experiment, its desired outcomes, and their beliefs about the effects of odours, as recommended by e.g., Howard and Hughes [88].

False beliefs about odour presence were not related to participants’ odour awareness or olfactory acuity (r ≤ 0.1, *p*s ≥ 0.4). This might be due to the fact that this sort of study is likely to attract volunteers who are rather olfaction-oriented and our participants moreover had to meet the requirement of normosmia. The absence of a significant link between false beliefs on the one hand and odour awareness and olfactory acuity on the other hand could therefore be attributed to low variation in the data. It is moreover also possible that individuals with greater odour awareness tend to generate more false alarms. False beliefs about the ambient atmosphere might also be due to the way in which we collected the data, that is, by explicitly asking participants about odour presence. This may have caused them to be more attentive to smells on subsequent awakenings. On the other hand, data exploration suggested that participants were not more likely to report smells as they progressed through the study and the nocturnal and the morning awakenings did not differ in terms of frequency of odour reports.

After controlling for other effects, including the participants’ appraisal of the ambient atmosphere, actual exposure did not significantly increase the likelihood of reporting chemosensory dreaming. It is unclear to what extent this may have been due to a prolonged exposure to olfactory stimuli, which may have led to olfactory adaptation. Habituation, its behavioural manifestation, could not be ruled out because less than half of the volunteers were able to detect the odour on either awakening and the percentage dropped over the course of exposure (63.3% and 44.8% for vanillin and 32.1% and 17.9% for thioglycolic acid on the first and second awakening, respectively). Of interest is the fact that thioglycolic acid seemed to have greater habituation effects than vanillin, while based on existing literature on olfactory habituation we expected the opposite [89,90]. On the other hand, very low direct and indirect incorporation rates have been observed even by researchers who used olfactometers to prevent olfactory adaptation and/or habituation [16,18,19]. For instance, Schredl, et al. [18] reported that of the 28 awakenings carried out upon exposure to either a generally pleasant odour (*n* = 13) or an unpleasant one, only four reports contained features which may have been related to olfactory stimulation, while in the control condition there was one explicit mention. In a study by Schredl, Hoffmann, Sommer, and Stuck [19], exposure to a generally pleasant odour produced a single report (out of 16 awakenings) rated as having an olfactory element, as did the control condition (*n* = 16), while stimulation with an unpleasant odour (*n* = 15) generated none. Okabe, Fukuda, Mochizuki-Kawai, and Yamada [16] found no explicit mention of olfactory elements in reports from 13 awakenings. Odour exposure nevertheless produced two reports that potentially involved chemosensory content (as judged by raters).

Another study found no effects of a continuous all-night odour exposure on dream reports. This was despite the fact that the volunteers were consciously aware of the stimulation [29]. In that study, researchers explicitly instructed their participants (12 insomniacs) to spray odorants onto their pillowcase and thus expose themselves to each odour for the period of four nights. It seems therefore that the lingering presence of an odour after awakening is unlikely to prompt reports of chemosensory dreaming. This is relevant to the current study because some participants may have used odour detection in the waking state as a cue to guide their reports of chemosensory content in sleep mentation. This could have involved any smells which participants believed to have noticed, because they were queried on any odours perceived in the room, potentially including any background smells. Yet we found no significant effect of participants’ appraisal of actual olfactory environment on their reports; the size of the observed effect fell well below what is deemed a ‘practically’ significant effect in psychological research [85].

The only factor which significantly increased the probability of reporting chemosensory dreaming was odour awareness, here operationalised as the OAS score. Its link with reports of chemosensory content in sleep mentation was previously observed by Stevenson and Case [40] in a retrospective questionnaire study. These researchers noted that relative to people whose MSEs did not contain smells, olfactory dreamers scored higher on the scale measuring interest in odours [91], thought that olfaction was more important to their occupation, considered themselves better smellers, and relied more on their sense of smell in everyday life, all of which could be subsumed under the broad term of odour awareness [42]. In the same vein, Arshamian, Willander and Larsson [43] postulated that higher rates of chemosensory content recall belong to manifestations of greater odour awareness. In line with this, Weitz, Croy, Seo, Negoias and Hummel [41] found that people who claimed to experience olfactory dream content were more olfaction-oriented than those who did not. The reasons underlying this phenomenon are unknown. One possibility is that greater reactivity to chemosensory stimuli during waking translates into more frequent chemosensory dream content. Another is that people who generally think of themselves as more olfaction-oriented tend to respond accordingly to any question concerning olfaction.

Stevenson and Case [40] did not report effect sizes but they could be calculated from the statistics: Cohen’s d [92] for the difference between olfactory dreamers and nondreamers in their interest in odours was 0.37, corresponding to *r* = 0.18. This means that there was an overlap in the scores of both the groups of about 84%, and a 61% chance that a person selected at random from the olfactory dreamer group would score higher than an olfactory nondreamer. Effect sizes for differences between the two groups in ratings of their sense of smell and its importance in everyday life were similar: d = 0.40 (*r* = 0.20) and d = 0.39 (*r* = 0.19), respectively. In the present study, we corroborated those findings. Pearson’s *r* for the link between reports of olfactory and gustatory content, respectively, and the OAS score ranged between 0.20–0.36 depending on the condition (odour/control) and awakening (1st/2nd). This corresponds to Cohen’s d of 0.41–0.77. Even if we take the highest value of d, that is, 0.77, it still gives an overlap of about 70% between those who reported chemosensory content in sleep mentation and those who did not, and about the same chance that a person picked at random from those whose sleep mentation contained smells (tastes) would score higher than an individual selected at random from those whose MSEs lacked such content. In sum, although odour awareness was a statistically significant factor contributing to chemosensory dreaming in the present study, its practical significance is limited.

Olfactory acuity, on the other hand, does not seem relevant to predictions of chemosensory content in sleep mentation. Although researchers who investigated olfactory dreaming using questionnaires [40,41] did report a statistically significant link, it should be noted that the effect sizes in their studies (as in the present one) were small. Stevenson and Case [40] used an unstandardised odour naming test consisting of fifteen household smells [93] and assessed free odour identification (odour naming). Effect sizes were not presented by the authors but can be calculated from the reported statistics: Cohen’s d = 0.47–0.48 (*r* = 0.23), depending on the method of scoring, which tends to be ambiguous for odour naming tests. Cohen’s d computed from descriptive statistics provided by Weitz, Croy, Seo, Negoias, and Hummel [41], who employed the Sniffin’ Sticks identification test [94], is 0.60 (*r* = 0.29). This means a 76.4% overlap between the scores of olfactory dreamers and nondreamers. Another way of interpreting this value is that there is only a 66.4% chance of someone selected at random from the olfactory dreamer group outperforming a person picked at random from the nondreamer group. The effect, while statistically significant, was thus small and barely practically significant [85]. In the present study, where we employed the standardised 40-item UPSIT [60], the effects of the odour identification ability on most awakenings were even smaller (*r* < 0.10) (but note the difference in how chemosensory dreaming was investigated).

An implication of the present findings is that reports of chemosensory content in sleep mentation seem to be linked to individual characteristics rather than to the present environment and the way it is perceived by the individual. Such characteristics may include a general tendency to notice and act upon olfactory stimuli. On the other hand, given that the whole model only explained less than 6% of variance in the outcome, there will be even more important factors that have not been addressed in the present study. For example, explorations of the data revealed that participants with higher BDI-II scores reported significantly more chemosensory dreams and that this effect was of medium size in those who recalled a dream on each awakening. This was despite the limited variation in depressive scores due to pre-screening (minimal depression range of 0–13 points) and *n* = 27. At the same time, there was a tendency for people with higher BDI-II scores to report less pleasant dreams. This is not a novel finding. There is some tentative evidence that heightened olfactory dreaming may accompany depressive episodes [95]. Studies on mentation involving smelling and tasting in various psychiatric disorders focus on chemosensory hallucinations rather than on dreaming. These, too, are relevant to the present discussion in that some may occur during the transition from waking to sleep or at awakening and may not be readily discernible from ‘dreams’ in the narrower sense of the word. For example, studies on representative samples of non-institutionalised general population from three European countries found that gustatory hallucinations tend to be associated with depressive disorders [96]. There is also evidence from patients with schizophrenia, schizoaffective disorder, and bipolar disorder that the rarer types of sensory hallucinations, including olfactory and gustatory ones, are correlated with a lifetime history of depressive episodes [97]. Future studies should thus further examine the link between chemosensory content in sleep mentation and depressed mood, both in the general population and in samples of persons with clinical depression, and identify the circumstances under which such experiences become unwanted and bothersome. Because the presence or absence of chemosensory content is more likely to be affected by a person’s general odour awareness than by the actual olfactory environment, subsequent studies should also investigate how such experiences could be modulated by altering a person’s reactivity to chemosensory stimuli.

An important limitation of the present study is that it was mostly university students who were available for repeated overnight polysomnographic assessments. The findings therefore cannot be generalised to the general population or even to just young adults. Although the initial pool of individuals interested in participation did include non-student population, the requirement that volunteers should not leave the laboratory before about 7 a.m. probably tended to collide with work schedules. Another problem was the scarcity of reports of chemosensory content, which made a quantitative analysis of the data a challenge. Although there exist sophisticated statistical tools capable of handling rare-event binary correlated outcomes in small samples [98], they are rather complicated for a non-statistician to implement. For the main analysis, we therefore employed GEE models, which can be used for binary correlated outcomes [77] but their estimates may be biased in samples consisting of less than 50 participants [99]. Given the cost of laboratory collection of sleep mentation, even doubling the sample size was not realistic in the present study. We therefore advise that the results of GEE modelling should be viewed with caution.

## 5. Conclusions

The present study sought to identify the factors which may affect reports of chemosensory (olfactory and gustatory) content in sleep mentation during all-night exposure. We found that the presence of smelling and tasting content in mental sleep activity was affected by individuals’ general odour awareness rather than by their current olfactory environment and its appraisal. This raises the question of whether and how such mental sleep experiences, if they become unwanted and bothersome, could be modulated by altering one’s reactivity to chemosensory stimuli in waking life.

## Figures and Tables

**Table 1:** Absolute and relative frequencies for education, time of going to bed, dream recall, and emotional intensity of dreams; and mean ± SD (range) for age, Beck Depression Inventory-II, Odor Awareness Scale, and University of Pennsylvania Smell Identification Test (‘odour identification’) scores, consumption of alcohol and stimulants, and usual sleep duration. Alcohol units for soft alcohol represent a small beer (0.33 L) or a glass of wine (0.2 L); for hard alcohol a 0.02 L glass. Units for coffee/tea and energy drinks are cups and cans, respectively. Gender differences were explored with Mann-Whitney U and Pearson chi-square tests. An asterisk (*) denotes a comparison for which the Freeman-Halton extension of the Fisher exact probability test for a two-rows by three-columns contingency table was performed.

	Men (*n* = 37)	Women (*n* = 23)	Total (*n* = 60)	Gender Difference			
				U	*p*	χ^2^(df)	*p*
Age	23.43 ± 4.24	23.43 ± 3.06	23.43 ± 3.80	387.0	0.556		
Education						3.21(2)	0.201 *
*Elementary*	1 (2.7%)	0	1 (1.7%)				
*Secondary*	25 (67.6%)	11 (47.8%)	36 (60%)				
*Bachelor’s Degree*	4 (10.8%)	6 (26.1%)	10 (16.7%)				
*Master’s Degree*	7 (18.9%)	6 (26.1%)	13 (21.7%)				
Beck Depression Inventory-II	3.92 ± 3.51 (0–11)	3.57 ± 3.16 (0–13)	3.78 ± 3.36 (0–13)	440.5	0.818		
Odor Awareness Scale	104.12 ± 14.59 (63–127)	113.30 ± 15.32 (83–143)	107.82 ± 15.43 (63–143)	275.5	0.060		
Odour identification	31.09 ± 4.11 (21–40)	31.30 ± 2.87 (25–37)	31.17 ± 3.64 (21–40)	396.5	0.924		
Alcohol (units/month)							
*Low-alcoholic drinks*	7.15 ± 6.06 (0–22)	4.39 ± 3.53 (0–16)	6.09 ± 5.38 (0–22)	536.5	0.091		
*Liquor*	1.92 ± 2.25 (0–10)	1.15 ± 1.95 (0–8)	1.63 ± 2.16 (0–10)	525.0	0.111		
Stimulants (units/month)							
*Coffee*	17.92 ± 25.35 (0–90)	16.74 ± 22.00 (0–60)	17.47 ± 23.94 (0–90)	400.0	0.695		
*Tea*	41.32 ± 46.11 (0–240)	61.57 ± 60.69 (0–240)	49.08 ± 52.62 (0–240)	334.5	0.164		
*Energy drinks*	1.32 ± 3.82 (0–20)	0.15 ± 0.46 (0–2)	0.88 ± 3.05 (0–20)	504.0	0.107		
*Cigarettes*	5.08 ± 16.20 (0–90)	0.57 ± 2.50 (0–12)	3.35 ± 12.93 (0–90)	495.5	0.115		
Time of going to bed on weekdays						0.01(1)	0.920
*Before midnight*	23 (62.16%)	14 (60.87%)	37 (61.67%)				
*At midnight or later*	14 (37.84%)	9 (39.13%)	23 (38.33%)				
Sleep duration (hours)							
*Weekdays*	7.33 ± 0.79 (6–9)	7.23 ± 0.70 (6–9)	7.30 ± 0.75 (6–9)	440.5	0.671		
*Weekends*	8.97 ± 0.91 (8–12)	8.75 ± 0.77 (7–10)	8.87 ± 0.86 (7–12)	459.0	0.461		
Dream recall						0.12(1)	0.729
*Once a week*	21 (56.76%)	12 (52.17%)	33 (55.00%)				
*More than once a week*	16 (43.24%)	11 (47.83%)	27 (45.00%)				
Emotional intensity of dreams						0.95(1)	0.329
*Somewhat intense*	24 (64.86%)	12 (52.17%)	36 (60.00%)				
*Quite or very intense*	13 (35.14%)	11 (47.83%)	24 (40.00%)				

**Table 2:** Absolute and relative frequencies for mental sleep experience (MSE) recall (yes/no), olfactory and gustatory content (present/absent) in the sample without the two outliers, sleep stage (REM vs. NREM), and belief about odour presence (present/absent). O1 and C1 refer to the first awakening in the odour and control condition, respectively, and O2 and C2 to the same upon the second awakenings. Mid-p tests (*p*-value, conditional maximum likelihood estimate of odds ratio [OR] with 95% confidence interval [95% CI], and *n*) were performed for paired observations in participants who could recall a ‘dream’ upon a particular awakening. *n*/A denotes analyses which were not feasible or meaningful due to zero cell counts.

	O1	C1	O2	C2	O1 vs. C1			O2 vs. C2			O1 vs. O2			C1 vs. C2		
					*p*	OR [95% CI]	*n*	*p*	OR [95% CI]	*n*	*p*	OR [95% CI]	*n*	*p*	OR [95% CI]	*n*
MSE recall	50/60 (83.3%)	49/60 (81.7%)	44/60 (77.2%)	43/60 (71.7%)	>0.900	1 [0.34, 2.98]	59	0.815	1.13 [0.42, 3.04]	55	0.302	1.8 [0.60, 5.93]	57	0.144	2.2 [0.78, 7.03]	57
Smell content	3/48 (6.3%)	3/47 (6.4%)	4/42 (9.5%)	2/41 (4.9%)	0.625	0.5 [0.02, 6.57]	40	0.625	2 [0.15, 58.99]	31	>0.900	1 [0.10, 9.61]	37	N/A	N/A	35
Taste content	6/48 (12.5%)	3/47 (6.4%)	4/42 (9.5%)	4/41 (9.8%)	N/A	N/A	40	0.688	0.67 [0.08, 4.48]	31	0.219	4 [0.50, 98.98]	37	0.625	0.5 [0.02, 6.57]	35
Sleep stage					0.727	1.33 [0.28, 7.15]	51	0.701	0.86 [0.39, 1.87]	57	<0.001	31 [5.92, 638.8]	53	<0.001	6.8 [2.82, 19.61]	58
*N1*	0	1/58 (1.7%)	2/58 (3.4%)	1/59 (1.7%)												
*N2*	5/53 (9.4%)	5/58 (8.6%)	32/58 (55.2%)	32/59 (54.2%)												
*N3*	0	0	3/58 (5.2%)	2/59 (3.4%)												
*REM*	48/53 (90.6%)	52/58 (89.7%)	21/58 (36.2%)	24/59 (40.7%)												
Belief about odour	28/58 (48.3%)	13/56 (23.2%)	18/57 (31.6%)	11/58 (19%)	<0.001	14 [2.48, 298.9]	54	0.049	3 [1.00, 10.78]	55	0.011	9 [1.48, 198.9]	55	0.545	1.5 [0.41, 6.03]	55

**Table 3:** The odds ratio (OR) and its 95% confidence limits (CL), χ^2^ statistic and *p*-value for condition (odour/control), participants’ appraisal of the olfactory environment (smell reported/not reported), odour awareness operationalised as the Odor Awareness Scale (OAS) score, and the ability to identify odour assessed by the University of Pennsylvania Odor Identification Test (UPSIT).

	OR	95% CL	χ^2^	*p*
Condition	1.86	[0.69; 5.00]	1.50	0.22
Participants’ assessment	1.67	[0.61; 4.56]	1.01	0.31
Odour awareness	1.05	[1.02; 1.08]	13.38	<0.001
Odour identification	0.91	[0.78; 1.06]	1.55	0.21

## Data Availability

Data analysed with Generalised Estimating Equations are displayed in Supplementary Table S2.

## Supplementary Materials

The following are available online at https://www.mdpi.com/article/10.3390/brainsci11091225/s1, Table S1: An overview of studies with adults reporting chemosensory (olfactory or gustatory) content in mental sleep experiences (‘dreams’), Table S2: Data used in the Generalised Estimating Equations (GEEs) and subsequent explorations, presented in a long format, Table S3: Descriptive statistics and comparisons for vanillin and thioglycolic acid in the odour condition on the 1st (O1) and 2nd (O2) awakening, Table S4: Predicted probabilities of reporting chemosensory content on the first awakening in the control condition (C1) according to University of Pennsylvania Smell Identification Test (UPSIT) and Odor Awareness Scale (OAS) scores and appraisal of olfactory environment, Table S5: Predicted probabilities of reporting chemosensory content on the second awakening in the control condition (C2) according to University of Pennsylvania Smell Identification Test (UPSIT) and Odor Awareness Scale (OAS) scores and appraisal of olfactory environment, Table S6: Predicted probabilities of reporting chemosensory content on the first awakening in the odour condition (O1) according to University of Pennsylvania Smell Identification Test (UPSIT) and Odor Awareness Scale (OAS) scores and appraisal of olfactory environment, Table S7: Predicted probabilities of reporting chemosensory content on the second awakening in the odour condition (O2) according to University of Pennsylvania Smell Identification Test (UPSIT) and Odor Awareness Scale (OAS) scores and appraisal of olfactory environment.
