# Supplementary material for: Olfaction-Related Factors Affecting Chemosensory Dream Content in a Sleep Laboratory
Source: Brain Sci. 2021 Sep 16;11(9):1225. doi: 10.3390/brainsci11091225 (PMC8465492; doi:10.3390/brainsci11091225)
Supplement: Supplementary file 1 [file brainsci-11-01225-s001.zip › brainsci-1302479-supplementary.pdf]

Supplementary Table S1

Olfaction-related factors affecting chemosensory dream content in a sleep laboratory

Lenka Martinec Nováková[a,b,*], Monika Kliková[a,c], Eva Miletínová[a,c], Jitka Bušková[a,c]

[a] *National Institute of Mental Health, Topolová 748, 250 67 Klecany, Czech Republic*

[b] *Department of Psychology and Life Sciences, Faculty of Humanities, Charles University, Pátkova 2137/5, 182 00 Prague 8, Czech Republic*

[c] *3rd Faculty of Medicine, Charles University, Ruská 87, 100 00 Prague 10, Czech Republic*

* Corresponding author. E-mail: lenka.novakova@nudz.cz, lenka.novakova@fhs.cuni.cz

Supplementary Table S1. An overview of studies with adults reporting chemosensory (olfactory or gustatory) content in mental sleep experiences ('dreams'). Studies involving collection of REM experiences are marked with an asterisk. N/A = not available or applicable

Author(s)	Sample	Laboratory assessment	Odour exposure	Dream assessment	Dream recall/record frequency	Chemosensory content (frequency, description)
Calkins (1893)	The author (28 yrs) and a male aged 32 yrs	No	No	Dream diary	Author: 205 dreams recorded over 55 nights, male: 170 dreams recorded over 46 nights	Olfactory: none<br>Gustatory: 2 (<1.0%)
Weed and Hallam (1896) with the assistance of E. D. Phinney	The authors, Phinney, and 4 students (referred to as "others")	No	No, except experimental stimulation in circa 10 cases (Hallam)	Dream diary	Weed: 141, Hallam: 150, Phinney: 50, others: 40 dreams recorded over 5-6 weeks	Weed: olfactory 21 (15.0%), gustatory 17 (12.0%)<br>Hallam: olfactory 4 (2.7%), gustatory 4 (2.7%)<br>Phinney: olfactory 1 (2.0%), gustatory 2 (4.0%)<br>Others: olfactory 0, gustatory 1 (2.5%)
Andrews (1900)	The author	No	Yes, stimulation with "intense and heavy odours" in an undisclosed number of cases	Dream diary	118 dreams/6 weeks	2 reports of olfactory content (1.7%) and 1 of gustatory one (<1.0%): smelling chloroform, ocean odour, drinking poisoned water
Monroe (1905)	55 female students	No	No	Dream diary kept for 6 successive mornings	287 dreams/6 mornings (mean 5.2 dreams)	2 reports of olfactory content (<1.0%) and 3 of gustatory one (1.0%)
McCormick et al. (1997)*	4 patients after right anatomical or functional hemispherectomy (1 M, 22.2 yrs, SD N/A), 8 controls (2 M, age N/A)	Nocturnal polysomnography for 3 consecutive nights (N1, N2 denote night 1, 2 respectively)	No	N1, N2: reports collected 5, 10, 15, and 20 min. after onset of 2nd, 3rd, 4th, and 5th REM respectively. Free recall of dreams (presence = report of 5 words or more) was followed by prompts for presence of characters, emotions, and sensory modalities.	Mean recall on N1: 84.6% for patients vs. 94.1% for controls; N2: 76.4% for patients vs. 92.3% for controls. N1: 1, 2, 3, or 4 dreams recalled by 0, 2, 1, and 1 patient and 3, 2, 3, and 0 controls respectively. N2: 1, 2, 3, or 4 dreams recalled by 0, 1, 1, and 2 patient and	N1: 0, 1, 2, 3 olfactory content reported by 2, 1, 1, 0 patients and 6, 2, 0, 0 controls; N2: 0, 1, 2, 1 patients and 7, 1, 0, 0 controls, respectively

Author(s)	Sample	Laboratory assessment	Odour exposure	Dream assessment	Dream recall/record frequency	Chemosensory content (frequency, description)
					0, 3, 2, 3 controls respectively.	
Zadra, Nielsen, and Donderi (1998)	49 male (33.2±13.5 yrs) and 115 female (35.5±13.8 yrs) participants	No	No	Retrospective accounts of olfactory and gustatory dreams Obtained with a Sleep/ Dream Questionnaire. Participants also kept dream diaries for 14-21 consecutive nights. Dreams then scored by researchers for "unambiguous" references to olfactory, gustatory and other sensory content.	Questionnaire: N/A<br>Dream diary: participants reported a mean of 20.6±10.8 dreams each (total=3372). Men: 18.3±8.9 dreams, women: 21.5±11.3 dreams, gender difference NS.	Questionnaire: olfactory content reported by 39.0% of participants (34.7% and 40.9% of men and women respectively); gustatory by 36.6% of participants (32.7% and 38.3% of men and women respectively). Gender differences NS.<br>Dream diaries: olfactory component identified in 1.0% of dreams (men: 0.1%, women: 1.3%), gustatory in 0.9% of dreams (men: 0.8%, women: 0.9%)
Happe, Klosch, and Zeitlhofer (2004)	17 myasthenia gravis outpatients (4 M, 49.5±13.6, 25-76 yrs), 14 healthy participants (4 M, 50.7±16.0, 23-75 yrs)	No	No	Dream questionnaire completed every morning for 2 weeks (235 nights/17 patients, 196 nights/14 controls)	Patients reported a mean of 6.8 dreams each (49.4% of nights, total=116), controls (37.2% of nights, total=73). Women: 48.0% of nights, men: 33.0% of nights<br>Two patients and 3 controls did not remember any dream.	15 dreams with an olfactory or gustatory component reported by patients, none by controls
Stevenson and Case (2004)	Study 1: 284 participants (78 M), age 16-24: 222, 25-34: 29, 35-44: 10, 45+: 23	No	No	Questionnaire assessing frequency of dream recall, presence and frequency of sensory content in dreams and its vividness. Participants were also asked to recall a dream in which the specific modality	Mean frequency of dream recall N/A but 280/284 (98.6%) reported having experienced visual dreams (=most frequent sensory modality)	Chemosensory dreaming reported by 90/284 (31.7%): 75 olfactory, 15 gustatory with an olfactory component, 16 both. An example dream provided by 49/75 (65.3%) of olfactory dreamers and 36/64 (56.3%) of gustatory dreamers, most often: eating and drinking or food

Author(s)	Sample	Laboratory assessment	Odour exposure	Dream assessment	Dream recall/record frequency	Chemosensory content (frequency, description)
				featured, describe it, indicate its most vivid component and rate it for hedonic tone and duration.		smells (51.3%), smoke and burning odours (21.3%), personal and animal odours (21.3%), and environmental (6.3%).
Okada, Matsuoka, and Hatakeyama (2005)	531 undergraduate students (318 M, 19.9, SD N/A, 18-36 yrs)	No	No	Questionnaire assessing frequency of various sensory experiences in dreams in the past month	N/A	Olfactory: 2.8% always, 6.2% fairly often, 12.0% occasionally, 34.5% seldom, 44.4% never Gustatory: 4.9% always, 10.7% fairly often, 11.6% occasionally, 37.3% seldom, 35.4% never
Zanasi, De Persis, Caporali, and Siracusano (2005)	148 healthy participants over 70 yrs of age (75.88±8.42 yrs), 151 controls (22.45±3.23, 18-25 yrs); proportion of gender N/A	No	No	Participants were asked to recount the last dream they could recall and their account was tape-recorded and transcribed	Equal to N due to study design	Smell featured in 1 elderly participant's dream (<1%), a taste dream reported by 1 younger participant (<1%)
Zanasi, Pecorella, Chiaramonte, Niolu, and Siracusano (2008)	100 patients with non-psychotic major depression (40 M, 42.8±4.4 yrs) and 250 healthy participants (147 M, 40.4±3.23 yrs)	No	No	Participants were asked to recount the last dream they could recall and their account was tape-recorded and transcribed	Equal to N due to study design	Smell featured in dreams of 7 controls (2.8%) and 2 patients (2.0%); 1 taste dream reported by 1 patient (1.0%) and no controls
Schredl et al. (2009)*	15 healthy women (23.0±2.1, range 20-28 yrs)	Nocturnal polysomnography for 2 consecutive nights (N2=night 2)	During N2, 4 ppm hydrogen disulphide (H_2S) or 20% v/v phenyl ethyl alcohol (PEA), odourless control (N/A) presented for 10 s. using an olfactometer. Stimulation	Reports (free recall) collected 1 min. after stimulation; presence of chemosensory modality assessed by 2 independent raters (criteria: mention of odour or elements "normally" associated with strong odour)	N2: 39/40 (97.5%) of awakenings; N1: N/A	N2: No explicit mention of olfactory dream content upon exposure to H_2S or PEA, 1/12 (8.3%) in odourless condition (smelling something rotten); no dreams rated by judges as having elements potentially associated with odour in the odourless condition, 13.3% and 15.4% upon stimulation with

Author(s)	Sample	Laboratory assessment	Odour exposure	Dream assessment	Dream recall/record frequency	Chemosensory content (frequency, description)
			occurred 5, 10, and 15 min. after onset of 1st, 2nd, 3rd, and each next REM respectively.			H_2S and PEA respectively (cleaning a toilet full of yellow liquid, eating a Kiwi fruit and eating potatoes with parsley, preparing a salad that included tuna, rice, corn and onions, and being in a stuffy room)
Weitz, Croy, Seo, Negoias, and Hummel (2010)	Study 1: 696 participants (252 M, 41.2±18.1, 11-85 yrs, 20 participants did not report age)	No	No	Questionnaire assessing frequencies of dream occurrence, recall, olfactory and/or gustatory content. Participants were also asked to give an example of an olfactory or gustatory dream.	N/A	Chemosensory dream content: 50/696 (7.2%) olfactory only, 44 (6.3%) gustatory only, 114 (16.4%) both; 110 women (24.8%) vs. 64 men (25.4%), gender difference NS. 105 participants provided at least one example of an olfactory or gustatory dream, resulting in 136 olfaction-related accounts. Most frequently reported were dreams involving food and drink (58), natural (28), and personal (22) odours, followed by burning smells (15) and odours of specific places (6).
Arshamian, Willander, and Larsson (2011)	20 healthy individuals scoring the highest on VVIQ, VOIQ and OIQ (5 M, 27.0±6.1, 19-41 yrs) selected from 45 olfactory/gustatory dreamers; 20 scoring the lowest (7 M, 26.2±7.3, 20-47 yrs) selected from 74 olfactory/gustatory non-dreamers	No	No	Retrospective dream reports for each sensory modality using the modified version of the Dream questionnaire (Stevenson & Case, 2004)	N/A	45/119 (37.8%) participants

Author(s)	Sample	Laboratory assessment	Odour exposure	Dream assessment	Dream recall/record frequency	Chemosensory content (frequency, description)
Kahan and LaBerge (2011)	16 participants (6 M, 20-47 yrs): 8 researchers (mean age=36 yrs) and 8 undergraduates (mean age=23 yrs); data from 1 researcher and 2 students excluded from analysis; SD not given	No	No	REM dreams recorded for 2 weeks (REM identified with DreamLight) Subjective Experiences Rating Scale to assess the prevalence (0 "none" to 4 "a lot") of eleven sensory and eleven affective qualities	Mean dream recall frequency based on self-reports: 5.43/week in men and 4.27/week in women (5-6 dreams/week in the total sample) Dream recall frequency based on the number of actually recorded dreams: 84.0% of the sleep samples	Mean prevalence ratings for olfactory and gustatory content in REM dreams were 0.47±0.69 and 0.26±0.43 respectively.
Voss, Tuin, Schermelleh-Engel, and Hobson (2011)	10 congenitally deaf-mute (4 M, 20.33 yrs, SE=2.96), 4 congenitally paraplegic individuals (all female, age N/A), 36 non-handicapped controls (2 M, 23.86 yrs, SE=1.0)	No	No	Dream diary kept for 2 weeks, participants asked to indicate presence of sensory content and its intensity	Deaf-mute, paraplegic, and control participants reported on average 8.71 (SE=1.67), 7.00 (SE=1.87), and 7.34 (SE=0.78) dreams respectively.	Deaf-mute, paraplegic, and control participants reported olfactory content in 4.93% (SE=2.76), 0%, and 10.45% (SE=3.61) of their dreams respectively.
Zanasi, Calisti, Di Lorenzo, Valerio, and Siracusano (2011)	123 stabilised schizophrenic inpatients (60 M, 31.9±8.9, 17-53 yrs; mean illness duration 13.2±3.4 yrs), 123 healthy participants (60 M, 31.18±4.90, 18-50 yrs)	No	No	Inpatients asked every morning if they had a dream last night (number of times each patient was approached N/A). If so, one of their dreams was tape-recorded and transcribed. (Details on data collection in healthy participants N/A.) Reports analysed by 2 independent raters.	Out of 173 patients approached 123 could recall a dream (71.1%; data on healthy participants N/A)	Smell featured in dreams of 4 controls (3.3%) and none of the patients; no dreams involving taste
Mota-Rolim et al. (2013)	3,427 respondents (24.0% M, 56.0% F, 20.0% N/A; median 25 yrs)	No	No	Online survey (details N/A, supplementary material N/A)	34.1% of respondents once or twice a week, 33.2% almost every day, 19.8% about twice a month, 9.2%	Odours and tastes/flavours reported by 20.0% and 26.8% of respondents, respectively

Author(s)	Sample	Laboratory assessment	Odour exposure	Dream assessment	Dream recall/record frequency	Chemosensory content (frequency, description)
					every day, 3.4% once a year, 0.1% less than once a year	
Lovati et al. (2014)	148 migraineurs: 66 with aura (MA; 16 M, 38.4±15.5 yrs), 82 without aura (M0; 19 M, 37.4±14.6 yrs); 45 patients with tension type headache (TTH; 15 M, 37.9±15.4 yrs); 219 controls (sex proportion and mean age N/A)	No	No	Semi-structured retrospective self-reported ad hoc questionnaire (details N/A)	MA: 59 (89.4%), M0: 73 (89.0%), TTH: 41 (91.1%), controls: 189 (86.3%)	Olfactory (% of participants, % of dreamers): MA: 24 (36.0%, 40.8%), M0: 29 (35.0%, 39.7%), TTH: 9 (20.0%, 22.0%), controls: 43 (20.0%, 22.8%) Gustatory: MA: 27 (40.9%, 45.8%), M0: 19 (23.2%, 26.0%), TTH: 5 (11.1%, 12.2%), controls: 43 (19.6%, 22.8%)
Meaidi, Jennum, Ptito, and Kupers (2014)	11 congenitally blind (CB, 5 M, 42±15 yrs), 14 late blind (LB, 7 M, 45±11 yrs), 25 sighted participants (12 M, 44±12 yrs)	No	No	Dreams noted every morning over 4 weeks; presence and clarity of olfactory and gustatory content	N/A	Olfactory: CB: 40%±36% of dreams, LB: 19%±17%, controls: 15%±25% Gustatory: CB: 26%±28% of dreams, LB: 11%±16%, control: 7%±18%
Schredl, Hoffmann, Sommer, and Stuck (2014)*	16 healthy participants (8 M, 23.5±3.8, 18-34 yrs) reporting dream recall frequency of at least once a week	Nocturnal polysomnography for 2 consecutive nights (N2=night 2)	Prior to N2: exposure to 4 ppm hydrogen disulphide (H_2S) or 20% v/v phenyl ethyl alcohol (PEA) using an olfactometer; N2: re-exposure to H_2S, PEA and odourless control in a balanced order for 10 s. Stimuli presented 5, 10, 15, and 15 min. after	Reports (free recall) collected 1 min. after stimulation; presence of chemosensory modality assessed by 2 independent raters	100%	2 dreams rated as olfactory: (1) smelling rose odour in a control (odourless) condition; (2) upon exposure to PEA: dreaming about taking part in an experiment in which the task was to differentiate between persons by smelling them

Author(s)	Sample	Laboratory assessment	Odour exposure	Dream assessment	Dream recall/record frequency	Chemosensory content (frequency, description)
			onset of 1st, 2nd, 3rd, and 4th REM respectively.			
Vitinius et al. (2014)	27 normosmic female inpatients (31.7±7.3, 20-49 yrs) suffering from mild to severe depression	Yes, but no polysomnography	Makeshift olfactometer: 50-ml bulb with room air and a cotton pad with 0.2 ml of PEA/clean pad; air puffs every 2 min. Exposure from sleep onset to 6 a.m.	Purpose-built questionnaire on dream characteristics, dream recall, sensory content	N/A but he frequency of recalled dreams did not differ between the conditions	N/A
Kahan and Claudatos (2016)	144 female undergraduates (19.35±3.24 yrs)	No	No	Dream diary (75 and 69 participants recorded dreams for 14 and 10 days respectively); Subjective Experiences Rating Scale to assess the prevalence (0 "none" to 4 "a lot") of eleven sensory and eleven affective qualities	458 dreams/2 weeks (of which 441 met word count criterion of ≥25 words); 351/10 days (of which 347 met the criterion)	Mean prevalence ratings for olfactory and gustatory experiences in awakening dreams were 0.50±1.01 and 0.66±1.17 respectively.
Okada and Wakasaya (2016)	86 students with hearing impairment (48 M, 16.6, 15-20 yrs, SD N/A), 344 hearing students (127 M, 16.7, 15-18 yrs, SD N/A)	No	No	Questionnaire developed for the purpose of the study assessing dream recall frequency (1=every day to 7=never), vividness, frequency of sensory content (1=always to 5=never) and emotions	3.25±1.64 in hearing-impaired vs. 3.93±1.47 in hearing students	Frequency of olfactory and gustatory content was 3.76±1.32 and 3.61±1.34 in hearing-impaired students; 4.13±1.03 and 4.13±1.02 in hearing students respectively.
Giani, Casazza,	45 migraineurs: 17 with aura (MA, all female,	No	No	Ad-hoc questionnaire assessing dream recall	Mean recall (number of dreams/month) was 17.9	5 (29.4%) MA, 16 (57%) M0, and 15 (25.4%) controls

Author(s)	Sample	Laboratory assessment	Odour exposure	Dream assessment	Dream recall/record frequency	Chemosensory content (frequency, description)
Mariani, and Lovati (2017)	40.53±12.27, 24-59 yrs), 28 without aura (M0, 6 M, 37.86±15.87, 23-70 yrs); 59 healthy participants (29 M, 32.75±13.29, 20-76 yrs)			and presence of sensory content was completed at awakening for 30 consecutive days.	(60%) in MA, 21.9 (73%) in M0, and 15 (50%) in controls.	reported having experienced olfactory or gustatory dream content at least once in 30 days. MA, M0 and controls reported on average 0.6 (2%), 2.1 (7%), and 0.6 (2%) dreams with olfactory/gustatory content respectively. Proportion of such dreams to total dreams was 0.03, 0.09, and 0.04 in MA, M0, and controls respectively.
Okabe, Fukuda, Mochizuki-Kawai, and Yamada (2018)*	15 students (6 M, 19.87±1.19, 18-22 yrs) who either liked (N=7) or disliked the odour of phenyl ethyl alcohol (PEA)	Nocturnal polysomnography for 1 night	50% PEA in distilled water (DW) or DW (control) presented 10 min. after REM onset for 10 s. using passive olfactometry; no participant reported awareness of odour	Reports (free recall) collected about 11 min. after onset of 2nd, 3rd REM; presence of chemosensory modality assessed by 2 independent raters (criteria: mention of odour or elements "normally" associated with strong odour)	13/15 (86.7%) reported a dream during 2nd REM; N/A for 3rd REM	No explicit mention of olfactory dreams; 2 dreams rated as having elements potentially associated with odour (eating buckwheat noodles, attending a pop music concert with a dog) reported during odour condition
Ackerley, Croy, Olausson, and Badre (2019)	12 participants (4 M, 43±12 yrs) with chronic mild–moderate insomnia	No	"Deep Sleep" and "Oriental" from This Works, control	Assessment after 4-night exposure, 3-night washout period for each odour	N/A	Incorporation ratings for either odour or control did not differ from the participants' typical dreams
Okabe, Hayashi, Abe, and Fukuda (2020)*	14 participants (4M, 18.9 ± 0.9 yrs) who were either highly familiar (N=7) or not with the odour of phenyl ethyl alcohol (PEA)	Nocturnal polysomnography for 1 night	See Okabe et al. (2020); those who noticed stimulation excluded from analyses	Dream ratings (Emotional Tone Scale, Dream Property Scale) and reports collected about 11 min. after onset of 2nd and later REMs	N/A	N/A

References

Ackerley, R., Croy, I., Olausson, H., & Badre, G. (2019). Investigating the putative impact of odors purported to have beneficial effects on sleep: neural and perceptual processes. *Chemosensory Perception,* Online First.

Andrews, G. A. (1900). Studies of the Dream Consciousness. II. *The American Journal of Psychology,* 12(1), 131-134.

Arshamian, A., Willander, J., & Larsson, M. (2011). Olfactory awareness is positively associated to odour memory. *Journal of Cognitive Psychology,* 23(2), 220-226.

Calkins, M. W. (1893). Statistics of dreams. *The American Journal of Psychology,* 5(3), 311-343.

Giani, L., Casazza, R., Mariani, C., & Lovati, C. (2017). Sensory modalities during dreams in migraine: case-control study using a daily questionnaire. *Dreaming,* 27(3), 260-268.

Happe, S., Klosch, G., & Zeitlhofer, J. (2004). Perception of dreams and subjective sleep quality in patients with myasthenia gravis. *Neuropsychobiology,* 50(1), 21-27.

Kahan, T. L., & Claudatos, S. (2016). Phenomenological features of dreams: Results from dream log studies using the Subjective Experiences Rating Scale (SERS). *Consciousness and Cognition,* 41, 159-176.

Kahan, T. L., & LaBerge, S. P. (2011). Dreaming and waking: Similarities and differences revisited. *Consciousness and Cognition,* 20(3), 494-514.

Lovati, C., DeAngeli, F., D'Amico, D., Giani, L., D'Alessandro, C. M., Zardoni, M., et al. (2014). Is the brain of migraineurs "different" even in dreams? *Neurological Sciences,* 35(1), 167-169.

McCormick, L., Nielsen, T., Ptito, M., Hassainia, F., Ptito, A., Villemure, J. G., et al. (1997). REM sleep dream mentation in right hemispherectomized patients. *Neuropsychologia,* 35(5), 695-701.

Meaidi, A., Jennum, P., Ptito, M., & Kupers, R. (2014). The sensory construction of dreams and nightmare frequency in congenitally blind and late blind individuals. *Sleep Medicine,* 15(5), 586-595.

Monroe, W. S. (1905). Mental elements of dreams. *The Journal of Philosophy, Psychology and Scientific Methods,* 2(24), 650-652.

Mota-Rolim, S., Targino, Z., Souza, B., Blanco, W., Araujo, J., & Ribeiro, S. (2013). Dream characteristics in a Brazilian sample: an online survey focusing on lucid dreaming. *Frontiers in Human Neuroscience,* 7(836).

Okabe, S., Fukuda, K., Mochizuki-Kawai, H., & Yamada, K. (2018). Favorite odor induces negative dream emotion during rapid eye movement sleep. *Sleep Medicine,* 47, 72-76.

Okabe, S., Hayashi, M., Abe, T., & Fukuda, K. (2020). Presentation of familiar odor induces negative dream emotions during rapid eye movement (REM) sleep in healthy adolescents. *Sleep Medicine,* 66, 227-232.

Okada, H., Matsuoka, K., & Hatakeyama, T. (2005). Individual differences in the range of sensory modalities experienced in dreams. *Dreaming,* 15(2), 106-115.

Okada, H., & Wakasaya, K. (2016). Dreams of hearing-impaired, compared with hearing, individuals are more sensory and emotional. *Dreaming,* 26(3), 202-207.

Schredl, M., Atanasova, D., Hoermann, K., Maurer, J. T., Hummel, T., & Stuck, B. A. (2009). Information processing during sleep: the effect of olfactory stimuli on dream content and dream emotions. *Journal of Sleep Research,* 18(3), 285-290.

Schredl, M., Hoffmann, L., Sommer, J. U., & Stuck, B. A. (2014). Olfactory stimulation during sleep can reactivate odor-associated images. *Chemosensory Perception,* 7(3-4), 140-146.

Stevenson, R. J., & Case, T. I. (2004). Olfactory dreams: phenomenology, relationship to volitional imagery and odor identification. *Imagination, Cognition and Personality,* 24(1), 69-90.

Vitinius, F., Hellmich, M., Matthies, A., Bornkessel, F., Burghart, H., Albus, C., et al. (2014). Feasibility of an interval, inspiration-triggered nocturnal odorant application by a novel device: a patient-blinded, randomised crossover, pilot trial on mood and sleep quality of depressed female inpatients. *European Archives of Oto-Rhino-Laryngology,* 271(9), 2443-2454.

Voss, U., Tuin, I., Schermelleh-Engel, K., & Hobson, A. (2011). Waking and dreaming: Related but structurally independent. Dream reports of congenitally paraplegic and deaf-mute persons. *Consciousness and Cognition,* 20(3), 673-687.

Weed, S. C., & Hallam, F. M. (1896). A study of the dream-consciousness. *American Journal of Psychology,* 7, 405-411.

Weitz, H., Croy, I., Seo, H. S., Negoias, S., & Hummel, T. (2010). Studies on Olfactory Dreaming. *Chemosensory Perception,* 3(2), 129-134.

Zadra, A. L., Nielsen, T. A., & Donderi, D. C. (1998). Prevalence of auditory, olfactory, and gustatory experiences in home dreams. *Perceptual and Motor Skills,* 87(3), 819-826.

Zanasi, M., Calisti, F., Di Lorenzo, G., Valerio, G., & Siracusano, A. (2011). Oneiric activity in schizophrenia: Textual analysis of dream reports. *Consciousness and Cognition,* 20(2), 337-348.

Zanasi, M., De Persis, S., Caporali, M., & Siracusano, A. (2005). Dreams and age. *Perceptual and Motor Skills,* 100(3), 925-938.

Zanasi, M., Pecorella, M., Chiaramonte, C., Niolu, C., & Siracusano, A. (2008). Dreams by persons with mood disorders. *Psychological Reports,* 103(2), 381-394.

Supplementary Table S2

Olfaction-related factors affecting chemosensory dream content in a sleep laboratory

Lenka Martinec Nováková[a,b,*], Monika Kliková[a,c], Eva Miletínová[a,c], Jitka Bušková[a,c]

[a] *National Institute of Mental Health, Topolová 748, 250 67 Klecany, Czech Republic*

[b] *Department of Psychology and Life Sciences, Faculty of Humanities, Charles University, Pátkova 2137/5, 182 00 Prague 8, Czech Republic*

[c] *3rd Faculty of Medicine, Charles University, Ruská 87, 100 00 Prague 10, Czech Republic*

* Corresponding author. E-mail: lenka.novakova@nudz.cz, lenka.novakova@fhs.cuni.cz

Supplementary Table S2. Data used in the Generalised Estimating Equations (GEEs) and subsequent explorations, presented in a long format. ID = participant no., cond = condition (1 = exposure, 0 = control), awak = awakening (1 = first, 2 = second), UPSIT = University of Pennsylvania Smell Identification Test score, OAS = Odor Awareness Scale score, CSd = chemosensory dream (1 = reported, 0 = not reported), odour = participants' appraisal of the olfactory environment (1 = odour reported, 0 = not reported), BDI = Beck Depression Inventory II score. Dots denote missing values.

ID	cond	awak	UPSIT	OAS	CSd	odour	BDI
01	0	1	29	117	.	0	4
01	0	2	29	117	0	0	4
01	1	1	29	117	.	1	4
01	1	2	29	117	.	1	4
02	0	1	28	114	.	.	1
02	0	2	28	114	.	0	1
02	1	1	28	114	1	1	1
02	1	2	28	114	0	0	1
03	0	1	26	107	.	0	7
03	0	2	26	107	0	0	7
03	1	1	26	107	0	1	7
03	1	2	26	107	0	1	7
04	0	1	26	95	0	1	4
04	0	2	26	95	.	1	4
04	1	1	26	95	0	0	4
04	1	2	26	95	.	0	4
05	1	1	33	98	0	0	5
05	1	2	33	98	0	0	5
05	0	1	33	98	0	0	5
05	0	2	33	98	.	.	5
06	1	1	31	88	0	0	1
06	1	2	31	88	0	0	1
06	0	1	31	88	0	0	1
06	0	2	31	88	0	0	1
07	1	1	21	.	0	1	0

07	1	2	21	.	0	0	0
07	0	1	21	.	0	0	0
07	0	2	21	.	.	0	0
08	0	1	26	117	0	0	0
08	0	2	26	117	0	0	0
08	1	1	26	117	1	0	0
08	1	2	26	117	0	0	0
09	0	1	31	116	0	0	6
09	0	2	31	116	0	0	6
09	1	1	31	116	1	1	6
09	1	2	31	116	0	1	6
10	0	1	34	111	.	0	2
10	0	2	34	111	.	0	2
10	1	1	34	111	1	1	2
10	1	2	34	111	.	0	2
11	1	1	27	66	.	.	0
11	1	2	27	66	0	0	0
11	0	1	27	66	0	0	0
11	0	2	27	66	.	0	0
12	1	1	36	140	0	1	3
12	1	2	36	140	0	1	3
12	0	1	36	140	1	1	3
12	0	2	36	140	0	1	3
13	0	1	36	108	0	0	1
13	0	2	36	108	0	0	1
13	1	1	36	108	0	0	1
13	1	2	36	108	0	0	1
14	0	1	37	117	.	1	10
14	0	2	37	117	0	0	10
14	1	1	37	117	0	1	10
14	1	2	37	117	0	0	10

15	0	1	27	110	0	0	13
15	0	2	27	110	1	1	13
15	1	1	27	110	0	1	13
15	1	2	27	110	0	0	13
16	0	1	34	110	0	0	5
16	0	2	34	110	.	0	5
16	1	1	34	110	0	0	5
16	1	2	34	110	0	0	5
17	1	1	31	123	0	1	1
17	1	2	31	123	0	1	1
17	0	1	31	123	0	0	1
17	0	2	31	123	0	1	1
18	1	1	24	100	1	0	8
18	1	2	24	100	0	0	8
18	0	1	24	100	1	0	8
18	0	2	24	100	0	0	8
19	1	1	30	101	0	0	0
19	1	2	30	101	0	0	0
19	0	1	30	101	0	0	0
19	0	2	30	101	0	0	0
20	1	1	37	111	0	0	6
20	1	2	37	111	.	0	6
20	0	1	37	111	0	0	6
20	0	2	37	111	0	0	6
21	0	1	33	118	0	0	7
21	0	2	33	118	0	0	7
21	1	1	33	118	1	0	7
21	1	2	33	118	1	0	7
22	1	1	29	106	0	1	4
22	1	2	29	106	0	1	4
22	0	1	29	106	0	1	4

22	0	2	29	106	0	1	4
23	1	1	31	109	0	0	4
23	1	2	31	109	0	0	4
23	0	1	31	109	0	0	4
23	0	2	31	109	.	0	4
24	1	1	32	112	0	1	2
24	1	2	32	112	.	.	2
24	0	1	32	112	0	1	2
24	0	2	32	112	0	1	2
25	1	1	36	105	0	1	0
25	1	2	36	105	0	1	0
25	0	1	36	105	0	1	0
25	0	2	36	105	0	1	0
26	0	1	32	110	1	0	3
26	0	2	32	110	1	0	3
26	1	1	32	110	.	1	3
26	1	2	32	110	.	1	3
27	0	1	35	122	1	1	11
27	0	2	35	122	.	0	11
27	1	1	35	122	1	1	11
27	1	2	35	122	1	0	11
28	1	1	32	114	0	0	0
28	1	2	32	114	0	0	0
28	0	1	32	114	0	0	0
28	0	2	32	114	0	0	0
29	1	1	37	128	0	0	2
29	1	2	37	128	0	0	2
29	0	1	37	128	0	0	2
29	0	2	37	128	0	0	2
30	1	1	32	111	0	0	3
30	1	2	32	111	0	0	3

30	0	1	32	111	0	0	3
30	0	2	32	111	0	0	3
31	1	1	34	100	.	0	9
31	1	2	34	100	.	0	9
31	0	1	34	100	.	0	9
31	0	2	34	100	.	0	9
32	1	1	29	100	0	1	1
32	1	2	29	100	1	1	1
32	0	1	29	100	0	0	1
32	0	2	29	100	0	0	1
33	0	1	35	81	.	.	10
33	0	2	35	81	0	0	10
33	1	1	35	81	0	0	10
33	1	2	35	81	0	0	10
34	0	1	31	102	0	0	1
34	0	2	31	102	0	0	1
34	1	1	31	102	0	0	1
34	1	2	31	102	0	0	1
35	1	1	31	63	0	1	2
35	1	2	31	63	0	0	2
35	0	1	31	63	0	0	2
35	0	2	31	63	0	0	2
36	0	1	29	106	0	1	2
36	0	2	29	106	0	0	2
36	1	1	29	106	.	1	2
36	1	2	29	106	0	1	2
37	0	1	32	123	.	0	4
37	0	2	32	123	1	0	4
37	1	1	32	123	0	0	4
37	1	2	32	123	0	0	4
38	1	1	32	93	0	0	2

38	1	2	32	93	0	0	2
38	0	1	32	93	0	0	2
38	0	2	32	93	0	0	2
39	0	1	.	88	0	1	3
39	0	2	.	88	.	0	3
39	1	1	.	88	.	1	3
39	1	2	.	88	.	.	3
40	1	1	25	83	0	0	0
40	1	2	25	83	0	0	0
40	0	1	25	83	0	0	0
40	0	2	25	83	0	0	0
41	1	1	33	.	.	1	0
41	1	2	33	.	.	0	0
41	0	1	33	.	0	1	0
41	0	2	33	.	0	0	0
42	0	1	34	127	0	0	2
42	0	2	34	127	0	0	2
42	1	1	34	127	0	0	2
42	1	2	34	127	.	.	2
43	1	1	31	98	0	0	0
43	1	2	31	98	.	0	0
43	0	1	31	98	0	0	0
43	0	2	31	98	0	0	0
44	0	1	30	98	0	0	2
44	0	2	30	98	0	1	2
44	1	1	30	98	0	0	2
44	1	2	30	98	0	0	2
45	0	1	27	93	0	1	6
45	0	2	27	93	0	0	6
45	1	1	27	93	0	1	6
45	1	2	27	93	.	1	6

46	0	1	30	106	0	0	2
46	0	2	30	106	1	0	2
46	1	1	30	106	0	1	2
46	1	2	30	106	0	0	2
47	1	1	40	111	0	1	11
47	1	2	40	111	.	1	11
47	0	1	40	111	0	0	11
47	0	2	40	111	.	0	11
48	0	1	35	107	.	.	0
48	0	2	35	107	.	.	0
48	1	1	35	107	.	0	0
48	1	2	35	107	0	0	0
49	0	1	29	110	.	0	7
49	0	2	29	110	.	0	7
49	1	1	29	110	0	.	7
49	1	2	29	110	.	0	7
50	1	1	30	106	.	0	7
50	1	2	30	106	1	0	7
50	0	1	30	106	0	0	7
50	0	2	30	106	0	0	7
51	1	1	29	125	0	0	5
51	1	2	29	125	0	0	5
51	0	1	29	125	0	0	5
51	0	2	29	125	0	0	5
52	0	1	30	98	0	0	6
52	0	2	30	98	.	0	6
52	1	1	30	98	.	1	6
52	1	2	30	98	1	1	6
53	1	1	26	115	0	0	8
53	1	2	26	115	0	0	8
53	0	1	26	115	0	0	8

53	0	2	26	115	0	0	8
54	0	1	31	107	0	0	3
54	0	2	31	107	.	1	3
54	1	1	31	107	0	0	3
54	1	2	31	107	1	0	3
55	0	1	32	.	0	0	1
55	0	2	32	.	0	0	1
55	1	1	32	.	0	1	1
55	1	2	32	.	0	1	1
56	0	1	31	117	0	0	6
56	0	2	31	117	0	0	6
56	1	1	31	117	1	0	6
56	1	2	31	117	.	0	6
57	1	1	31	98	0	1	0
57	1	2	31	98	.	1	0
57	0	1	31	98	.	.	0
57	0	2	31	98	0	0	0
58	0	1	33	142	0	0	7
58	0	2	33	142	.	0	7
58	1	1	33	142	1	0	7
58	1	2	33	142	0	1	7

Supplementary Table S3

Olfaction-related factors affecting chemosensory dream content in a sleep laboratory

Lenka Martinec Nováková[a,b,*], Monika Kliková[a,c], Eva Miletínová[a,c], Jitka Bušková[a,c]

[a] *National Institute of Mental Health, Topolová 748, 250 67 Klecany, Czech Republic*

[b] *Department of Psychology and Life Sciences, Faculty of Humanities, Charles University, Pátkova 2137/5, 182 00 Prague 8, Czech Republic*

[c] *3rd Faculty of Medicine, Charles University, Ruská 87, 100 00 Prague 10, Czech Republic*

* Corresponding author. E-mail: lenka.novakova@nudz.cz, lenka.novakova@fhs.cuni.cz

Supplementary Table S3. Descriptive statistics and comparisons for vanillin and thioglycolic acid in the odour condition on the 1st (O1) and 2nd (O2) awakening. N (%) is given for the participants who reported they could smell an odour. M = mean, T = Wilcoxon signed rank test statistic, U = Mann-Whitney U; effect sizes: *r* = Pearson's *r*, OR = odds ratio. Statistically significant findings ($p < 0.05$) are highlighted in bold. Within-subject comparisons for thioglycolic acid were not meaningful because N = 4.

	Vanillin							Thioglycolic acid				Vanillin vs. Thioglycolic acid					
	O1		O2		O1 vs. O2			O1		O2		O1			O2		
	N = 19 (63.3%)		N = 13 (44.8%)		N = 13			N = 9 (32.1%)		N = 5 (17.9%)		19 vs. 9 (p = .018, OR = 3.65)			13 vs. 5 (p = .028, OR = 3.74)		
	M	SD	M	SD	T	p	r	M	SD	M	SD	U	p	r	U	p	r
Pleasantness	6.68	1.97	6.54	2.07	<.01	.083	-0.34	3.56	1.13	3.40	1.52	14.5	**<.001**	.67	7.0	**.010**	.61
Intensity	5.63	2.27	5.54	2.33	<.01	**.041**	-0.40	4.11	2.15	4.20	1.92	53.0	.105	.31	21.5	.271	.26
Familiarity	6.00	2.54	6.23	1.92	5.50	.140	-0.29	5.00	1.73	4.60	2.51	60.5	.214	.23	16.5	.109	.38

Supplementary Tables S4 - S7

Olfaction-related factors affecting chemosensory dream content in a sleep laboratory

Lenka Martinec Nováková[a,b,*], Monika Kliková[a,c], Eva Miletínová[a,c], Jitka Bušková[a,c]

[a] *National Institute of Mental Health, Topolová 748, 250 67 Klecany, Czech Republic*

[b] *Department of Psychology and Life Sciences, Faculty of Humanities, Charles University, Pátkova 2137/5, 182 00 Prague 8, Czech Republic*

[c] *3rd Faculty of Medicine, Charles University, Ruská 87, 100 00 Prague 10, Czech Republic*

* Corresponding author. E-mail: lenka.novakova@nudz.cz, lenka.novakova@fhs.cuni.cz

Supplementary Table S4. Predicted probabilities of reporting chemosensory content on the first awakening in the control condition (C1) according to University of Pennsylvania Smell Identification Test (UPSIT) and Odor Awareness Scale (OAS) scores and appraisal of olfactory environment. The scores for the given percentile are shown in parentheses.

		Odour not reported					Odour reported				
		UPSIT percentile					UPSIT percentile				
		10th (26)	25th (29)	50th (31)	75th (34)	90th (36)	10th	25th	50th	75th	90th
OAS percentile	10th (88)	4%	3%	3%	2%	2%	14%	12%	10%	9%	7%
	25th (98)	6%	5%	4%	4%	3%	23%	19%	17%	14%	12%
	50th (108)	10%	8%	7%	6%	5%	33%	29%	26%	22%	19%
	75th (116)	15%	12%	11%	9%	8%	44%	38%	35%	31%	27%
	90th (124)	21%	17%	15%	13%	11%	54%	49%	45%	41%	36%

Supplementary Table S5. Predicted probabilities of reporting chemosensory content on the second awakening in the control condition (C2) according to University of Pennsylvania Smell Identification Test (UPSIT) and Odor Awareness Scale (OAS) scores and appraisal of olfactory environment. The scores for the given percentile are shown in parentheses.

		Odour not reported					Odour reported				
		UPSIT percentile					UPSIT percentile				
		10th (26)	25th (29)	50th (31)	75th (34)	90th (36)	10th	25th	50th	75th	90th
OAS percentile	10th (88)	10%	9%	8%	6%	5%	17%	14%	12%	11%	9%
	25th (98)	13%	11%	9%	8%	7%	20%	17%	15%	13%	11%
	50th (108)	16%	13%	12%	10%	8%	24%	21%	19%	16%	14%
	75th (116)	18%	15%	14%	12%	10%	28%	24%	22%	19%	16%
	90th (124)	21%	18%	16%	14%	12%	32%	28%	25%	22%	19%

Supplementary Table S6. Predicted probabilities of reporting chemosensory content on the first awakening in the odour condition (O1) according to University of Pennsylvania Smell Identification Test (UPSIT) and Odor Awareness Scale (OAS) scores and appraisal of olfactory environment. The scores for the given percentile are shown in parentheses.

		Odour not reported					Odour reported				
		UPSIT percentile					UPSIT percentile				
		10th (26)	25th (29)	50th (31)	75th (34)	90th (36)	10th	25th	50th	75th	90th
OAS percentile	10th (88)	8%	5%	3%	2%	1%	12%	7%	5%	3%	2%
	25th (98)	17%	10%	7%	4%	3%	23%	14%	10%	6%	4%
	50th (108)	32%	21%	15%	10%	6%	41%	28%	20%	13%	9%
	75th (116)	48%	34%	25%	17%	11%	57%	42%	33%	23%	15%
	90th (124)	64%	49%	39%	28%	19%	72%	58%	48%	36%	25%

Supplementary Table S7. Predicted probabilities of reporting chemosensory content on the second awakening in the odour condition (O2) according to University of Pennsylvania Smell Identification Test (UPSIT) and Odor Awareness Scale (OAS) scores and appraisal of olfactory environment. The scores for the given percentile are shown in parentheses.

		Odour not reported					Odour reported				
		UPSIT percentile					UPSIT percentile				
		10th (26)	25th (29)	50th (31)	75th (34)	90th (36)	10th	25th	50th	75th	90th
OAS percentile	10th (88)	14%	16%	16%	18%	19%	22%	23%	25%	26%	28%
	25th (98)	14%	15%	16%	17%	18%	22%	23%	24%	26%	27%
	50th (108)	14%	15%	16%	17%	18%	21%	23%	24%	26%	27%
	75th (116)	14%	15%	16%	17%	18%	21%	23%	24%	25%	27%
	90th (124)	14%	15%	16%	17%	18%	21%	22%	24%	25%	27%
